# T lymphocyte subpopulations and intestinal helminthes profile among tuberculosis patients co-infected with HIV before and after anti tubercular treatment at University of Gondar Hospital, Northwest Ethiopia

**DOI:** 10.1186/s12879-020-4845-y

**Published:** 2020-02-07

**Authors:** Tadelo Wondmagegn, Debasu Damtie, Meaza Genetu, Belete Biadgo, Mulualem Lemma, Markos Negash

**Affiliations:** 1 Department of Immunology and Molecular Biology, School of Biomedical and Laboratory Sciences, College of Medicine and Health Sciences, University of Gondar, Gondar, Ethiopia; 2 Department of Clinical Chemistry, School of Biomedical and Laboratory Sciences, College of Medicine and Health Sciences, University of Gondar, Gondar, Ethiopia

**Keywords:** Tuberculosis, TB/HIV co-infection, T lymphocyte sub population, Intestinal helminthes

## Abstract

**Background:**

Tuberculosis continues to be a health problem of both developed and developing countries, and its incidence has currently increased due to HIV induced immune suppression. HIV-co-infection decreases the total number of CD4+ T cells since the virus preferentially replicates with in activated CD4+ T cells and macrophages, resulting in the disruption of granuloma to contain M. tuberculosis. In this study, we investigated the change in T lymphocyte subpopulations before and after anti-tubercular treatment and the effect of intestinal parasites on the cell populations of tuberculosis patients before the initiation of anti TB treatment.

**Method:**

A prospective cohort study was conducted in the outpatient TB Clinic, University of Gondar hospital between January 2014 and August 2015. Blood samples were collected from 80 newly diagnosed TB patients with and without HIV co-infection. The mean CD4+ and CD8+ T lymphocyte counts of the patients were assessed before and after the course of anti-TB treatment. The mean values of T lymphocytes of TB, TB/HIV co-infected patients and of the control groups were compared. Data was analyzed by SPSS version 16 and the graph pad prism software.

**Results:**

A total of 80 tuberculosis patients 40 of whom were co-infected with HIV participated in our study. The mean CD4 + T lymphocytes counts of the TB/HIV cohort were 354.45 ± 138cell/μl, and the mean CD8+ cell counts were 926.82 ± 384cell/μl. There were significant changes in the mean CD4+ and CD8+ T cell counts after the course of anti-TB treatment in both groups of patients(*p* < 0.05). However, no statistically significant differences were observed in the mean CD4 + and CD8+ T cell counts of helminthes infected and non-infected patients (*P* > 0.05).

**Conclusion:**

We found significantly lower CD4+ T cell counts among TB infected HIV negative patients compared with controls who showed that TB was the cause of non–HIV-associated declination of circulating CD4 counts, and the reduction was reversible with anti-tubercular treatment in both HIV-negative and ART naïve TB-HIV co-infected patients.

## Background

Tuberculosis (TB) is one of the commonest causes of morbidity and mortality relating to infectious diseases, particularly in developing countries [1]. TB is transmitted via the inhalation of aerosolized droplets containing the bacilli of *Mycobacterium tuberculosis* [2]. The incidence of the disease has steadied or declined in most regions of the world. But, is rising in some parts like Africa, Southeast Asia, and the Eastern Mediterranean in association with conditions, like immunodeficiency due to HIV [3]. Ethiopia is a high TB burden country where tuberculosis remains a serious public health problem. According to WHO Global TB report 2017, Ethiopia ranked 7th among the high TB burden countries in the world, with an estimated incidence of 172 of all forms of new cases/100,000 population and 29,000 deaths in 2016. The estimated prevalence of TB in Ethiopia was reported as 209/100,000 population [4].

HIV-TB co-infection induces an overwhelming impact on tuberculosis control in Sub-Saharan Africa [5]. In countries with the highest HIV prevalence, more than 75% of the tuberculosis cases are HIV positive [6]. Of the 9.4 million new cases of active TB reported each year across the globe, 1.4 million of the victims are HIV-positive [7]. The HIV-TB co-infection is among the many factors which have been preventing high TB burden countries from attaining the global plan to end TB epidemic between 2016 and 2035 [4].

HIV depletes CD4+ T cells and changes CD8+ T cell counts which have essential roles in preventing clinical diseases following TB infection [8]. HIV also has marked effects on other cells, like macrophages and affects cytokine production which may disrupt the host immune system from containing the initial or latent *Mycobacterium tuberculosis* infection [9]. The cause for the disruption of the immune response is associated with a decrease in the total number of CD4+ T cells which are preferentially targeted by the HIV virus [10]. Conventionally, measurements of CD4+ and CD8+ T lymphocyte counts are used as common markers of the immune system, and declines in these cells as predictors of disease progression and mortality [11].

Moreover, it has been considered that helminthes infection may be associated with chronic immune activation, promoting a Th2 type of immune response in helminthes co-infected TB patients [12, 13]. However, studies showing that change in T-lymphocyte populations after tuberculosis treatment and the effects of intestinal helminthes infection on these cell populations among TB and TB/HIV co-infected patients are limited. Therefore, in the present study, we investigated the change in T lymphocyte subpopulations after anti-tubercular treatment and the effect of intestinal parasites on these cell populations before the initiation of tuberculosis treatments.

## Methods

### Study design, area, period and population

A prospective cohort study was conducted in the TB Outpatient clinic, University of Gondar hospital, Northwest Ethiopia, from January 2014 to August 2015. Adult TB patients with and without HIV co-infection who presented for anti-tubercular treatment in the TB outpatient clinic during the study, and a reference group of matched healthy controls were recruited from staff members of the University.

### Sample size and sampling technique

A total of 80 newly diagnosed TB patients (40 only TB & 40 TB-HIV co-infected) consecutively visiting the University of Gondar hospital TB Outpatient clinic, were included in this study. Age and sex-matched forty apparently healthy controls also took part. Baseline information, blood, and stool samples were collected from each participant. Blood samples used for repeating CD4+ and CD8 + T lymphocyte counts after 6 months were collected for a second time from the same patients after they had completed their anti-TB treatments.

### Data collection and processing

#### Baseline data

Patients who fulfilled the inclusion criteria (TB patients naive to DOTs) and signed the written informed consent were enrolled. Data on socio-demographic characteristics were collected by the investigators using a pre-tested semi-structured questionnaire. Similarly, participants` heights and weights were measured to calculate the Body Mass Index (BMI). Baseline CD4+ and CD8+ T lymphocyte counts, stool examination, urine hCG (for females) and HIV testing were done for all study participants. HIV testing was carried out according to the national algorithm recommended by the Federal Ministry of Health of Ethiopia. Rapid HIV tests: HIV (1 + 2) rapid test strip (KHB Shanghai Kehua Bio-engineering Co, LTD, Shanghai, China) as a screening test; and Stat-Pak (Chembio Diagnostic Systems, Inc., New York, NY, USA) as a confirmatory test for positive samples; and Uni-Gold™ (Trinity Biotech Plc, Bray, Ireland) as a tie-breaker test were run. These HIV testing methods were immuno-chromatographic assays. All samples with non-reactive results to KHB were considered negative. Following the baseline assessment, patients were started on anti-tubercular treatment in accordance with the guidelines for the use of anti-tubercular drugs in Ethiopia, where, TB patients are put on a standard regimen of 2 months of isoniazid, rifampin, pyrazinamide, and ethambutol, followed by 4 months of isoniazid and rifampicin) [14].

#### Post-treatment data

The CD4+ and CD8+ T lymphocyte count of all of the participants were repeated after 6 months of follow up and completion of treatments.

### Sample collection and laboratory procedures

Four ml (4 ml) of blood sample was collected using a pre-labeled K3 EDTA vacutainer tube and the CD4+ and CD8+ T lymphocyte counts were done using the Multi TESTTM fluorescent labelled monoclonal antibody reagents against surface CD markers (CD3-flurescein isothiocyanate (FITC), CD8- phycoerythrin (PE) and CD4-allophycocyanin (APC) after erythrocyte has been lysed on the same day of sample collection using a fluorescent activated cell sorter (FACS Calibur, Becton Dickinson Immunocytometry System, San Jose, California, USA) at the University of Gondar hospital ART laboratory. About 1 mg of stool samples collected in a clean, dry leak proof screw top containers were transported to the parasitology laboratory to be examined for intestinal parasites using the wet mount and formol-ether concentration technique [15]. Urine specimens were collected from female participants in clean dry urine collection cups and immediately tested for Human Chorionic Gonadotropin Hormone (HCG) using the wondfo rapid One-Step HCG Test kit.

### Data management and analysis

The statistical analyses were performed using SPSS version 16 and the graph pad prism software. Data were analyzed in two steps. First, the data that assessed the frequency, the mean and the standard deviation of the baseline characteristics and a one way analysis of variance were used to test the normality of the data distribution. A second analysis was done using laboratory data collected before and after the initiation of ATT. Paired and Independent t-test was used to compare the means within a group and between groups. The Mann-Whitney U test was used to compare the non-parametrical data, and all reported *P*-value < 0.05 were considered as statistically significant.

## Results

### Socio-demographic and baseline characteristics

A total of 80, 52(65%) male and 28(35%) female, newly diagnosed TB patients in the age range of 18–55 years and a mean age of (33.08 ± 9.9) years participated in this investigation.. Most (52.5%) of the patients were illiterate. Over all 61, (76.25%) of the study participants had pulmonary and 19(23.75%) extra-pulmonary tuberculosis; 40 apparently healthy men and women were also took part as non-TB controls (Table 1).

**Table 1:** Socio-demographic characteristics of the study population from January 2014 and August 2015 at University of Gondar hospital TB outpatient clinic

	HIV^+^/TB patients (*n* = 40)	HIV^−^/TB patients (*n* = 40)	Healthy controls (*n* = 40)
Mean age (years)	35.15	32.27	31.82
Gender, n(%):			
Male	22 (55.0)	30 (75.0)	29 (72.5)
Female	18 (45.0)	10 (25.0)	11 (27.5)
Occupation, n(%):			
Student	6 (15.0)	5 (12.5)	6 (15.0)
Government employed	9 (22.5)	2 (5.0)	29 (72.5)
Daily laborer	5 (12.5)	7 (17.5)	2 (5.0)
Farmer	9 (22.5)	14 (35.0)	–
Merchant	3 (7.5)	1 (2.5)	2 (5.0)
Other	8 (20.0)	11 (27.5)	1 (2.5)
Residence, n(%):			
urban	26 (65.0)	21 (52.5)	39 (97.5)
Rural	14 (35.0)	19 (47.5)	2 (2.5)
Religion,n(%):			
Orthodox	37 (92.5)	35 (87.5)	32 (80.0)
Protestant	–	–	2 (5.0)
Muslim	3 (7.5)	5 (12.5)	6 (15.0)
Level of education, n(%):			
Illiterate	18 (45.0)	24 (60.0)	4 (10.0)
High school	12 (30.0)	12 (30.0)	4 (10.0)
Diploma	6 (15.0)	3 (7.5)	9 (22.5)
Degree	4 (10.0)	1 (2.5)	23 (57.5)

*NB* Other occupations include housewife, private employees and priest

Forty HIV un-infected TB and 40 HIV-TB co-infected patients enrolled in this study. The study revealed that there was a significant difference in the mean Body Mass Index (BMI) of apparently healthy controls and cases (*p* < 0.05), but there was no significant difference in the mean body mass index of HIV un-infected and HIV-TB co-infected patients (18.29 and 17.71, respectively) at baseline. However, the mean CD8 + T lymphocyte count of TB-HIV co-infected patients (926.82 ± 384cell/μl) was higher than that of the HIV un infected TB patients (711.12 ± 651 cell/μl), In fact, the difference (*p* = 0.075) was not statistically significant. The study also revealed that the mean CD8+ T lymphocyte count of the control groups was significantly lower than that of the TB-HIV co-infected patients (706.47 ± 38 cell/μl) (*p* = 0.003) (Table 2).

**Table 2:** Baseline CD4+ and CD8+ T lymphocyte count and IP status of study participants January 2014 to August 2015 at University of Gondar Hospital TB outpatient clinic

	HIV^+^/TB patients (*n* = 40)	HIV^−^/TB patients (*n* = 40)	Healthy controls (*n* = 40)
Type of TB:			
Pulmonary smear positive	5 (12.5)	15 (37.5)	–
Pulmonary smear negative	25 (62.5)	16 (40.0)	–
Extra pulmonary TB	10 (25.0)	9 (22.5)	–
Mean CD4 cell count (SD)	354.45 ± 138	637.15 ± 301	931.87 ± 44
Mean CD8 cell count (SD)	926.82 ± 384	711.12 ± 651	706.47 ± 38
Stool examination, n(%):			
Positive for IP	23 (57.5)	23 (57.5)	–
Negative for IP	17 (42.5)	17 (42.5)	–

*IP* Intestinal Parasite, *TB* Tuberculosis, *SD* Standard Deviation, *HIV* Human Immunodeficiency Virus, *CD4* Cluster of Differentiation 4

### Change in the mean CD4 + T lymphocyte counts of HIV-infected and uninfected TB patients

**Fig. 1:**
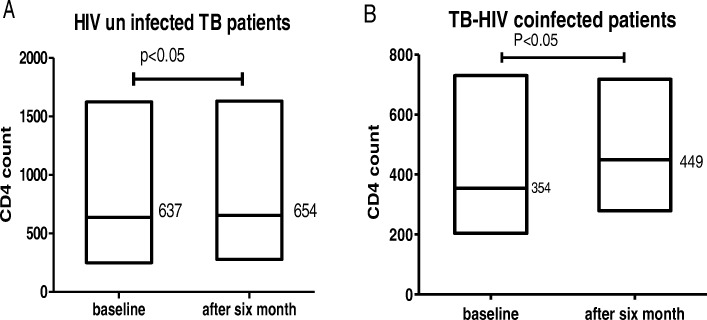
Progress of mean CD4+ T lymphocyte counts in HIV un infected (**a**) and HIV infected (**b**) TB patients after ATT, the figure shows mean CD4+ T lymphocyte at the baseline and at the end of TB treatment from January 2014 and August 2015 at University of Gondar Hospital TB outpatient clinic

The mean CD4+ T lymphocyte counts of HIV and HIV-TB co-infected patients showed a significant improvement after 6 months of anti-tubercular treatment follow ups. The CD4+ T lymphocyte count of HIV-TB co-infected patients increased from 354.45 ± 138 cell/μl to 449.15 ± 131 cell/μl, (*p* < 0.001), and among the HIV uninfected TB patients, the CD4+ T lymphocyte count increased from 637.15 ± 301 cell/μl to 654.07 ± 303 cell/μl (*p* < 0.001) (Fig. 1).

### Change in the mean CD8 + Tlymphocyte counts of HIV-infected and uninfected TB patients

**Fig. 2:**
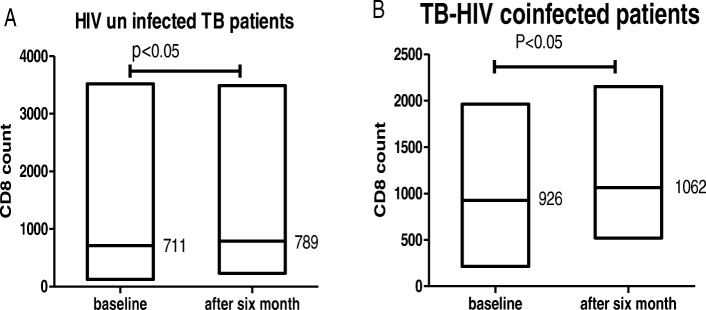
Progress of mean CD8+ T lymphocyte counts in HIV uninfected (**a**) and HIV infected (**b**) TB patients after ATT, the figure shows mean CD8+ T lymphocyte at the baseline and at the end of TB treatment from January 2014 and August 2015 at University of Gondar Hospital TB outpatient clinic

After tuberculosis treatment, the mean CD8+ T lymphocyte count of HIV-infected and uninfected TB patients increased significantly by rising from 926.82 ± 384 cell/μl to 1062.47 ± 381 cell/μl and 711.12 ± 651 to 789.27 ± 606 cell/μl (*p* < 0.001),respectively. (Fig. 2).

### Differences in the mean CD4 + and CD8 + T lymphocyte counts of TB patients with and without intestinal parasites

Both TB and TB-HIV co-infected patients were tested for intestinal parasite using the formol-ether concentration technique. The overall finding showed that 57.5%(46/80) of the patients were positive for at least one parasite. Hookworm and *Ascaris lumbricoid* accounted for 21.25(17/80) and 13.5% (11/80) of the commonly identified parasites in both groups of participants, respectively. Six participants were infected with more than one parasite. The mean CD4+ T lymphocyte counts of HIV uninfected TB patients with and without parasitic infections were 600.53cell/μl and 664.22 cell/μl, respectively. However, there was no significant difference in the mean CD4+ counts of these groups (*p* = 0.652). Similarly, a statistically significant difference was not observed in the mean CD4+ T lymphocyte counts among intestinal parasite infected and non-infected TB-HIV co-infected patients (334.74, 381.12) cell/μl respectively (*p* = 0.468) (Table 3).

**Table 3:** Baseline CD4 + and CD8+ count in IP Infected TB and TB-HIV co-infected patients from the beginning of January to the end of February 2014 at Gondar town TB clinics

	IP negative (*n* = 17)	IP positive (23)	*P* value
TB^+^ mean CD_4_	600.53	664.22	0.652
TB^+^-HIV^+^ mean CD_4_	381.12	334.74	0.468
TB^+^ mean CD_8_	808.53	639.13	0.256
TB^+^-HIV^+^ mean CD_8_	972.47	893.09	0.859

*IP* Intestinal Parasite, *TB* Tuberculosis, *SD* Standard Deviation, *HIV* Human Immunodeficiency Virus, *CD4* Cluster of Differentiation 4, *CD8* Cluster of Differentiation 8

## Discussion

Host defense against tuberculosis is mainly mediated by the cell-mediated immune response which involves the various subsets of lymphocytes, including the CD4 + and CD8 + T cells, and their depletion triggers host vulnerability to TB [16]. In this study, significant differences were noted in the mean CD4+ T lymphocyte counts of HIV infected and uninfected TB patients prior to anti TB treatment, with the mean CD4+ T lymphocyte counts of 354.45 ± 138 and 637.5 ± 301 cells/μl, respectively. The mean CD8+ T lymphocyte counts also varied as (926.82 ± 384 cells/μl and 711.12 ± 651 cells/μl) across the group, respectively. Our finding is supported by those of studies done in Guinea-Bissau and India [17, 18], which revealed marked differences in the mean CD4+ T lymphocyte counts of HIV infected and HIV free TB cases. In addition, significant differences similar to what were noted in our investigation were recorded in the mean CD8+ T lymphocyte counts in Guinea-Bissau and India studies [17, 18].

Extensive studies reported significant improvements in mean CD4+ T lymphocyte counts after patients received anti-tuberculosis treatments. A study done in Argentina reported a mean CD4+ T lymphocyte count increase from 314.25 to 413 cells/μl [19]; a similar study done in Karnataka, India, also showed the progress of mean CD4+ T lymphocyte count from 197 to 300 cells/μl [20]. Another study on TB-HIV co-infected patients in India revealed a rise in mean CD4+ cell count from 292 to 379 cells/μl after treatment, which is in line with our finding. In contrast, a study in New Delhi, Northern India, marked reduction (from 194 to 162 cells/μl and 593 to 232cells/μl) of mean CD4+ count among participants after they took treatment in TB and TB-HIV co infected patients respectively) [17]. The possible explanation for the observed reduction in CD4+ cell counts may be related to peripheral blood lymphocytopenia due to severe diseases as most patients in the Indian study were only chronic pulmonary TB patients. On the other hand, studies done in Uganda and India on HIV infected and uninfected TB patients demonstrated that there was no change (610 to 602 cells/μl) in the mean CD4+ count before and after treatment [21, 22].

In our study, the mean CD8+ T lymphocyte counts of our participants changed (significantly) from 711.12 ± 651 cells/μl to 789.27 ± 606 cells/μl in HIV uninfected and from 926.82 ± 384 cells/μl to 1062.47 ± 381 cells/μl in HIV co-infected TB patients after treatment. Our finding was inconsistent with those of Argentina (259 to 321 cells/μl) [19], and India (747 to 803cells/μl) in TB-HIV co-infected cases and (536 to 709) in HIV uninfected TB patients [17]. Likewise, a Saudi Arabian study revealed a significant change of CD8+ cell count from 1136 to1316.54 cells/μl after the completion of anti-tubercular treatment in HIV uninfected TB patients [11]. In contrast, reports from a Ugandan study showed that there was a significant reduction in the mean CD8+ count after treatment (1568 to 1226 cells/μl) [22]. Our finding differed from that of Uganda, perhaps due to the differences in the study population since the participants in the Ugandan study were patients with only pulmonary tuberculosis, while we included some (*n* = 19) extra-pulmonary tuberculosis cases.

Since helminthes infections promote a Th2 immune response, individuals with helminthes might be expected to have lower CD4 + T cell counts than those without [23]. The present finding on the effects of helminthes infection on the CD4+ and CD8+ T lymphocyte counts showed that there was no statistically significant difference in the mean CD4 + & CD8+ T cell counts in TB patients with and without intestinal parasite at baseline. The result is in agreement with that of a study done in Akaki, Ethiopia [24]. In contrast, a large prospective cohort of HIV-infected ART-naive patients in Uganda reported the presence of a statistically significant difference in the mean CD4+ and CD8+ cell counts of helminthes-infected and helminthes free TB patients. This discrepancy may be due to the fact that the patients in the Ugandan study had a higher parasitic load than the participants of our study. Variations in techniques of determining the frequency of immune cells may also matter, FACS count was used for analyzing CD4+ and CD8+ cell counts in the Ugandan study could have given rise to procedural and instrumental errors. As a drawback, TB diagnosis and treatment in this study was based on Ethiopian guidelines without any conformational tests (PCR, culture) other than smear microscopy. The study confirmed that all HIV-TB co-infected participants were ART naive at the beginning of the study, but we didn’t assess the effects and the adherence of patients on ART. That we used a limited number of study participants was our limitation, too.

## Conclusion

In conclusion, our study found a significant difference in the mean CD4+ and CD8+ T cell counts of HIV uninfected and TB-HIV co-infected patients before TB treatment. Moreover, in both cases the amount is lower compared to those of healthy controls, indicating TB is a cause of non HIV associated depletion of circulating CD4+ T cell counts, and this reduction is rescindable with anti-tubercular treatments. Besides, TB additionally contributes to a reduction in CD4+ counts in TB-HIV co-infected individuals; this would be potentially reversible by the administration of 6 months of ATT. this study has also gave some insight about the efficacy of ATT in improving the CD4+ and CD8+ cell counts in both HIV uninfected TB patients and ART naïve TB- HIV co-infected patients. The study also showed helminthes infection has no effect on mean CD4 + and CD8+ counts.

## Data Availability

Data regarding the finding of this manuscript is contained in the result section as well; additional data and materials are available up on request to the corresponding author.

## Abbreviations

AIDS: Acquired Immune Deficiency Syndrome
ATT: Anti Tubercular Treatment
BD: Becton Dickinson
BMI: Body Mass Index
DOTS: Directly Observed Treatment, Short-course
EDTA: Ethylene-Diamine Tetra Acetic acid
FACS: Fluorescent Activated Cell Sorter
HCG: Human Chorionic Gonadotropin
HIV: Human Immunodeficiency Virus
TB: Tuberculosis
